# Enhancement of the Device Performance and the Stability with a Homojunction-structured Tungsten Indium Zinc Oxide Thin Film Transistor

**DOI:** 10.1038/s41598-017-12114-y

**Published:** 2017-09-14

**Authors:** Hyun-Woo Park, Aeran Song, Dukhyun Choi, Hyung-Jun Kim, Jang-Yeon Kwon, Kwun-Bum Chung

**Affiliations:** 1 Division of Physics and Semiconductor Science, Dongguk University, Seoul, 100-715 Korea; 2 Department of Mechanical Engineering, School of Engineering, Kyung Hee University, Yongin, 446-701 Korea; 3 School of Integrated Technology, Yonsei University, Incheon, 406-840 Korea

## Abstract

Tungsten-indium-zinc-oxide thin-film transistors (WIZO-TFTs) were fabricated using a radio frequency (RF) co-sputtering system with two types of source/drain (S/D)-electrode material of conducting WIZO (homojunction structure) and the indium-tin oxide (ITO) (heterojunction structure) on the same WIZO active-channel layer. The electrical properties of the WIZO layers used in the S/D electrode and the active-channel layer were adjusted through oxygen partial pressure during the deposition process. To explain enhancements of the device performance and stability of the homojunction-structured WIZO-TFT, a systematic investigation of correlation between device performance and physical properties at the interface between the active layer and the S/D electrodes such as the contact resistance, surface/interfacial roughness, interfacial-trap density, and interfacial energy-level alignments was conducted. The homojunction-structured WIZO-TFT exhibited a lower contact resistance, smaller interfacial-trap density, and flatter interfacial roughness than the WIZO-TFT with the heterojunction structure. The 0.09 eV electron barrier of the homojunction-structured WIZO-TFT is lower than the 0.21 eV value that was obtained for the heterojunction-structured WIZO-TFT. This reduced electron barrier may be attributed to enhancements of device performance and stability, that are related to the carrier transport.

## Introduction

Amorphous-oxide semiconductor thin-film transistors (AOS-TFTs) have attracted much attention as the next-generation transparent and flexible/wearable electronics such as flat-panel displays^1, 2^, sensors^3^, smart windows^4^, and photovoltaic cells^5, 6^ because of superior effective mobility that is more than 10 cm^2^/Vs, a high transparency in the visible-light region that is more than 80%, and a low process temperature that provides performance superior to conventional amorphous silicon TFTs. In addition, they are also a lower-cost process, and offer better electrical uniformity than polysilicon TFTs because of their amorphous structure^7–9^. Despite various advantages of oxide semiconductors, an understanding of the interfacial state between the oxide-channel layer and the adjacent layers is necessary to design and realize high-performance TFT devices due to the stacked structure of actual TFT devices. Especially, one of the essential issues is that it is difficult to form sound electrical contacts between an oxide-semiconductor layer and source/drain (S/D) electrodes; to overcome this issue, a decreased S/D electrode resistivity is typically required. Accordingly, low-resistivity materials such as Mo^10^, Cu^11^, Al^11^, Au/Ti^12^, Pt/Ti^13^, indium tin oxide (ITO)^14^, and indium zinc oxide (IZO)^12^ are suitable for the S/D electrode. In addition, contact resistance between the semiconductor and the S/D electrode are other considerable factors for determination of a material and a process for electrodes. High-contact resistance may induce the current crowding at the output characteristics, and finally, the signal delay is increased even though the electrode-material resistivity is sufficiently low. Recently, several researchers have reported improvement methods regarding contact resistance between the semiconductor and the S/D electrode that include thermal annealing^15^, UV irradiation^16^; Ar-, He-, and H_2_-plasma treatment, and other treatments^17–19^. Unfortunately, these processes are complicated, and in particular, plasma treatment produces collision damage while hydrogen is diffused to the semiconductor layer, and both effects may adversely impact the electrical characteristic of TFT devices.

Since the oxide semiconductor can be used to adjust electrical characteristics according to oxygen partial pressure during the deposition process, the electrode layer as well as the semiconductor layer can be formed as one material through controlling of the oxygen partial pressure. More specifically, it has been reported that increasing of the oxygen partial pressure as an active channel layer during the oxide-semiconductor deposition shifts the electronic-transport behavior of the film from conductor-like to semiconductor-like^20–22^. Based on this result, it is possible to fabricate a homojunction-structured TFT device with low-contact resistance that is composed of one material by using a conducting layer as the electrodes and a semiconducting layer as the active channel layer.

Our previous study provided detailed results on the correlation between device performance and electronic structure of tungsten doped InZnO TFTs with respect to tungsten-doping concentration. In addition, we also suggested WIZO semiconductor material as a suitable active layer to solve the problem of bias instability because of the W element as excellent carrier suppressor, caused by its high oxygen bond dissociation energy. In this study, we provide a facile method to enhance device performance and stability of a WIZO thin film transistor using a homojunction structure with systemically analysis results in terms of the electronic structure, including aspects such as the contact resistance, surface/interfacial roughness, band-edge state below the conduction-band, refractive index, and energy level alignments.

## Experimental procedure

### Fabrication of the WIZO-TFTs with the homojunction and heterojunction structures

To fabricate the homojunction- and heterojunction-structured WIZO-TFT devices, a heavily doped *p*-type silicon (p^++^-Si) wafer with a resistivity from 0.001 Ω·cm to 0.005 Ω·cm was used as the back-gate electrode, and a 100-nm-thick SiO_2_ layer that served as gate dielectrics was thermally grown on the Si substrate for the creation of an inverted staggered-bottom-gate type with a top contact. Then, the WIZO active-channel layer was deposited with the use of a co-sputtering of WO_3_ and InZnO (1:1 at. %) as the sputtering target in a radio frequency (RF) sputtering system without substrate heating. eTo minimize variability of process conditions and contamination on the target surface, film deposition was started after a pre-clean sputtering of the target for 15 minutes. RF-power values of the WO_3_ and the InZnO target for established optimum concentration of the tungsten doping from a previous study were fixed at 10 W and 150 W, respectively, and the WIZO active-layer thickness is 10 nm^23^. Process pressure and relative oxygen-flow rate were set as 5 mTorr and the O_2_/(Ar + O_2_) ratio was 0.05, respectively. During the WIZO-film deposition, the active-layer region was defined using a shadow mask. Next, two types of 100-nm-thick S/D electrode were formed on the same WIZO active layer with repeated use of the shadow mask. First, the conducting WIZO S/D electrode was deposited with the same RF sputtering system as WIZO active layer without substrate heating. The only difference of conducting WIZO S/D electrode is the without oxygen gas provided during the deposition process. Also, to optimize deposition conditions of the conducting WIZO S/D electrode, tungsten-doping concentration were controlled from 0% to 8% through variation of the input RF power of the WO_3_ target from 5 W to 20 W while fixing that of the InZnO target at 150 W. Second, the ITO S/D electrode was deposited with the use of InSnO (9:1 at. %) as the sputtering target and the RF power, process pressure and the relative oxygen-flow rate during the deposition process were set as 100 W, 5 mTorr and 0%, respectively.

Structures of the fabricated homojunction (WIZO active layer-WIZO S/D electrode)- and heterojunction (WIZO active layer-ITO S/D electrode)-structured WIZO-TFTs are of the staggered-bottom-gate type, and the channel length (L) and the width (W) are 800 μm and 200 μm, respectively. Last, all WIZO-TFTs were annealed at 250 °C for one hour under an air atmosphere using a furnace system.

### Electrical and physical measurements

To optimize electrical resistivity of conducting WIZO S/D electrode as a function of the tungsten-doping concentration, Hall-effect measurements were conducted using van der Pauw configuration with the permanent magnet of 0.55 Tesla at room temperature. Transfer characteristics and hysteresis behavior of WIZO-TFT devices with two types of S/D electrode were measured at room temperature using the Keithley SCS-4200 semiconductor-parameter analyzer. During electrical measurements, drain-to-source voltage (*V*_*DS*_) was fixed at 10.1 V and the drain-to-source current (*I*_*DS*_) was measured through sweeping of the gate-to-source voltage (*V*_*GS*_) from −20 V to 20 V. To examine electrical contact between the S/D electrode and the WIZO active layer, the well-known transmission line method (TLM) was conducted. Changes of film density and surface/interfacial roughness between the S/D electrode and the WIZO active layer according to the different S/D electrodes were examined using atomic force microscopy (AFM) and X-ray reflectivity (XRR). Especially, the XRR experiment was conducted at the 10D beamline in the Pohang Accelerator Laboratory (PAL) in the Korea, and obtained XRR data were fitted using X’Pert Reflectivity software.

### Electronic-structure measurements and energy-level alignments

Electronic structures that are related to changes of the band-edge state below the conduction band and energy-level alignments were investigated using X-ray photoelectron spectroscopy (XPS), Kelvin probe force microscopy (KPFM), and spectroscopic ellipsometry (SE). In particular, SE measurement was conducted using a rotating analyzer system with an auto-retarder in the energy range from 0.74 eV to 6.4 eV, with the incident angles of 65°, 70°, and 75°. In addition, change of the interfacial states depending on the device structure was examined by Transmission Electron Microscopy (TEM) analysis.

## Results and Discussion

It has been reported that electrical and optical properties of the WIZO films are crucially affected by oxygen partial pressure during the sputtering-deposition process^20, 21^, as shown in S1 (Supplementary Information). To consider structural effects and chemical composition of WIZO films as a function of oxygen partial pressure during the deposition process, XRD and XPS were investigated as shown in S2 (Supplementary Information) and Table 1. Preferred orientations and their qualitative comparison are comparable, because all XRD spectra were measured by theta–2theta X-ray diffractometer and normalized by Si (400) from Si (100) substrate. Regardless of oxygen pressure during the deposition process, the diffraction pattern of WIZO films represent typical polycrystalline structure with diffraction peaks of ZnO (002), ZnO (102), ZnO (110), and In_2_O_3_ (440) except for peaks of Si substrate. These indicate that the WIZO films have polycrystalline and different oxygen pressure during the deposition process shows no effects on modification of the physical structure. However, chemical composition of the oxygen atom bounded to metal (W, In, Zn) of the conducting WIZO is reduced compared to semiconducting WIZO, which can generate free electrons related to oxygen deficient states. Based on this result, it is possible to fabricate a homojunction-structured TFT device that comprises a low contact resistance and is composed of one material. Since electrical properties of the WIZO layer were sensitively changed by tungsten-doping concentration as well as oxygen partial pressure^24^, electrical properties of the conducting WIZO-electrode layer were systematically optimized according to tungsten-doping concentration with the use of two procedures, as shown in S3 (Supplementary Information). First, electrical properties of conducting WIZO films as a function of tungsten-doping concentration were investigated using the Hall-effect measurement at room temperature. As the tungsten-doping doping concentration increased from 0% to 8%, carrier concentrations of the WIZO film gradually decreased from 5.2 × 10^20^/cm^3^ to 1.72 × 10^19^/cm^3^. However, as the tungsten-doping concentration increased from 0 to 4%, the hall mobility of WIZO film increased from 3.76 cm^2^/Vs to 12.9 cm^2^/Vs. Conversely, at tungsten-doping concentrations more than 4%, hall mobility of WIZO film significantly decreased to 0.03 cm^2^/Vs. Here, the decrement of carrier concentration is strongly associated with a decrement of the oxygen deficiency state caused by increase of doping concentration of tungsten with high oxygen bond dissociation energy. In addition, the parabolic trend of hall mobility with increasing tungsten doping concentration is resulted from the changes of electronic structure by incorporation of excess tungsten atoms, provided in the previous study^23^. At the same time, the conducting WIZO layer according to tungsten-doping concentration was formed as the S/D electrode layer on the optimized WIZO active-channel layer for evaluation of electrical characteristics of the TFT device. Both results reveal optimized electrical properties at tungsten-doping concentration of 4%; additionally, with the increasing of tungsten-doping concentration until −8%, resistivity of WIZO films and device performance of the WIZO-TFTs were degraded. Interestingly, the exhibited resistivity of the optimized tungsten-doped WIZO film is slightly lower than that of ITO film with values of 1.1 × 10^−3^ Ω·cm, and 3.5 × 10^−3^ Ω·cm. The homojunction-structured WIZO-TFT device reveals a small hysteresis-derived threshold-voltage shift compared with the heterojunction-structured WIZO-TFT device. Detailed explanation of the origin are subsequently provided for enhanced device performance and stability of the optimized homojunction-structured WIZO-TFT from comparison with the heterojunction-structured WIZO-TFT.

**Table 1:** Chemical compositions of the ITO, conduction WIZO, and semiconducting WIZO layers.

	W4f	Chemical composition (at. %)			
		In3d	Zn2p	Sn3d	O1s
ITO	—	53.1	—	5.3	41.6
Conducting WIZO	0.6	25.7	22.9	—	50.8
Semiconducting WIZO	0.4	24.8	19.8	—	55.1

**Figure 1:**
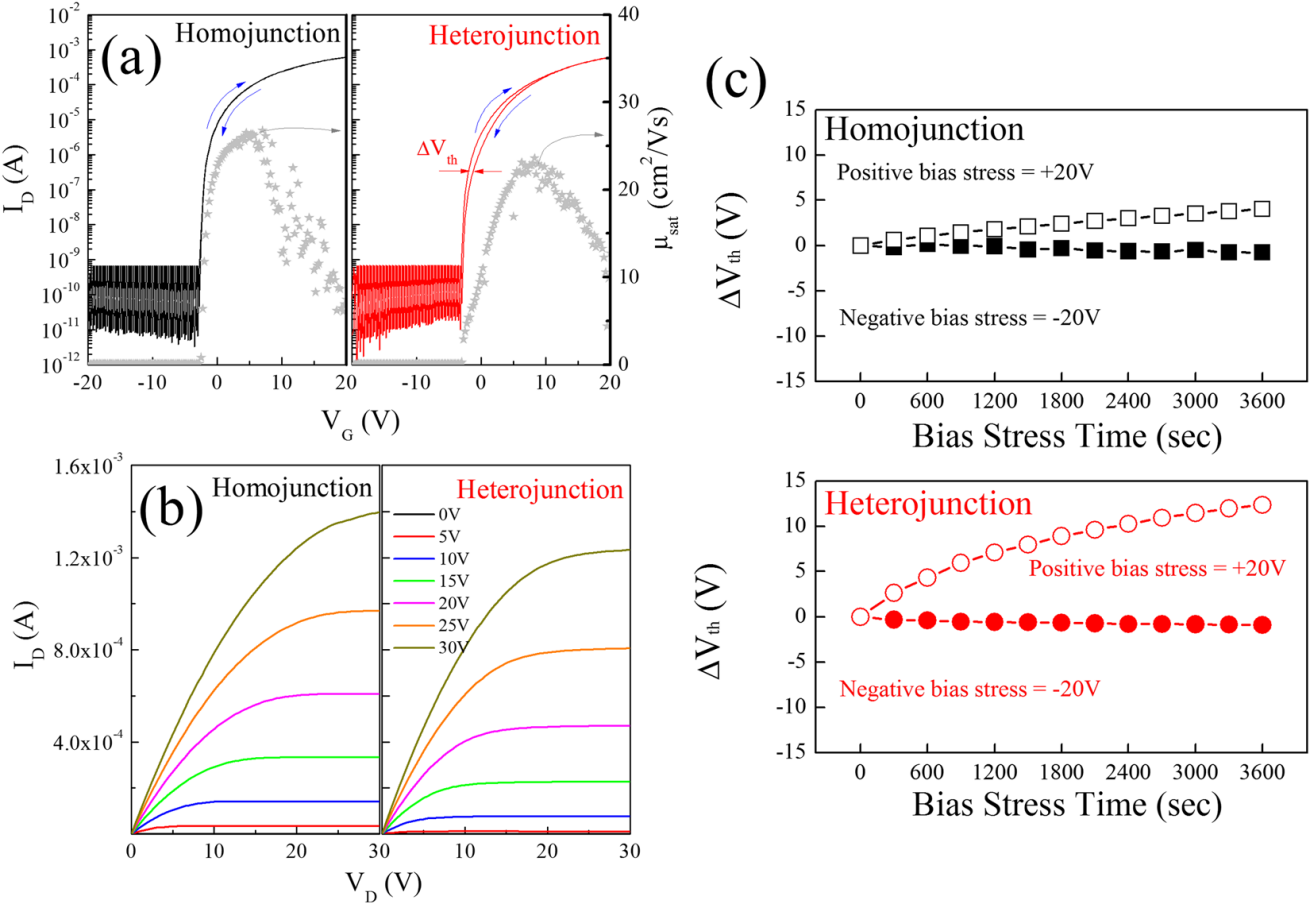
(**a**) Transfer, hysteresis, saturation mobility and (**b**) output characteristics of the WIZO-TFTs according to the S/D electrode materials, the conducting WIZO, and the indium-tin oxide (ITO) layer. (**c**) Shift of threshold voltage under positive and negative bias stress of WIZO TFTs according to the S/D electrode materials.

Figure 1(a) and (b) show the representative transfer, hysteresis, saturation mobility and output characteristics of WIZO-TFTs according to different S/D electrodes. Hysteresis behavior obviously reveals that the clockwise hysteresis is positively related to the S/D electrode, as shown in Fig. 1(a). The hysteresis-derived threshold-voltage shift (*ΔV*_*th*_) in the homojunction-structured WIZO-TFT is insignificant at 0.07 V, whereas the shift of 0.32 V is significant in the heterojunction-structured WIZO-TFT. Generally, the hysteresis characteristic is related to trap densities at the semiconductor and/or the interfacial region between the semiconductor and the gate insulator, dramatically enhanced in the homojunction-structured WIZO-TFT. This means that trap densities of the semiconductor and/or the interfacial region are affected by S/D electrode materials. The homojunction-structured WIZO-TFT exhibited enhanced device performance with saturation mobility (*μ*_*sat*_) and subthreshold gate-swing (*S.S*.) value, as well as excellent hysteresis behavior, compared with the heterojunction-structured WIZO-TFT. The detailed device parameters are summarized in Table 2. The bias instability of WIZO TFTs according to the two types of electrode was investigated under bias stress condition, with a negative and positive gate bias of −20 V and 20 V for 3600 s, as shown in Fig. 1(c). As the stress time passed, the V_th_ of the heterojunction-structured WIZO-TFT shifts by −0.91 V and 12.3 V, while that in the homojunction-structured WIZO-TFT exhibited only −0.71 V and 4.02 V shift in V_th_. These indicate that the type of electrode affects device stability.

**Table 2:** Device parameters of the WIZO-TFT, including the *V*_*th*_, *μ*_*sat*_, *S.S*., and the on/off current ratio according to the different S/D electrodes.

	*V*_*th*_ [V]	*ΔV*_*th, hysteresis*_ [V]	*μ*_*sat*_ [cm^2^/Vs]	*S.S*. [V/decade]	*I*_*ON/IOFF*_
Homojunction-structured WIZO-TFT	−3.35	0.07	26.10	0.38	7.39 × 10^6^
Heterojunction-structured WIZO-TFT	−2.80	0.32	21.02	0.44	6.42 × 10^6^

**Figure 2:**
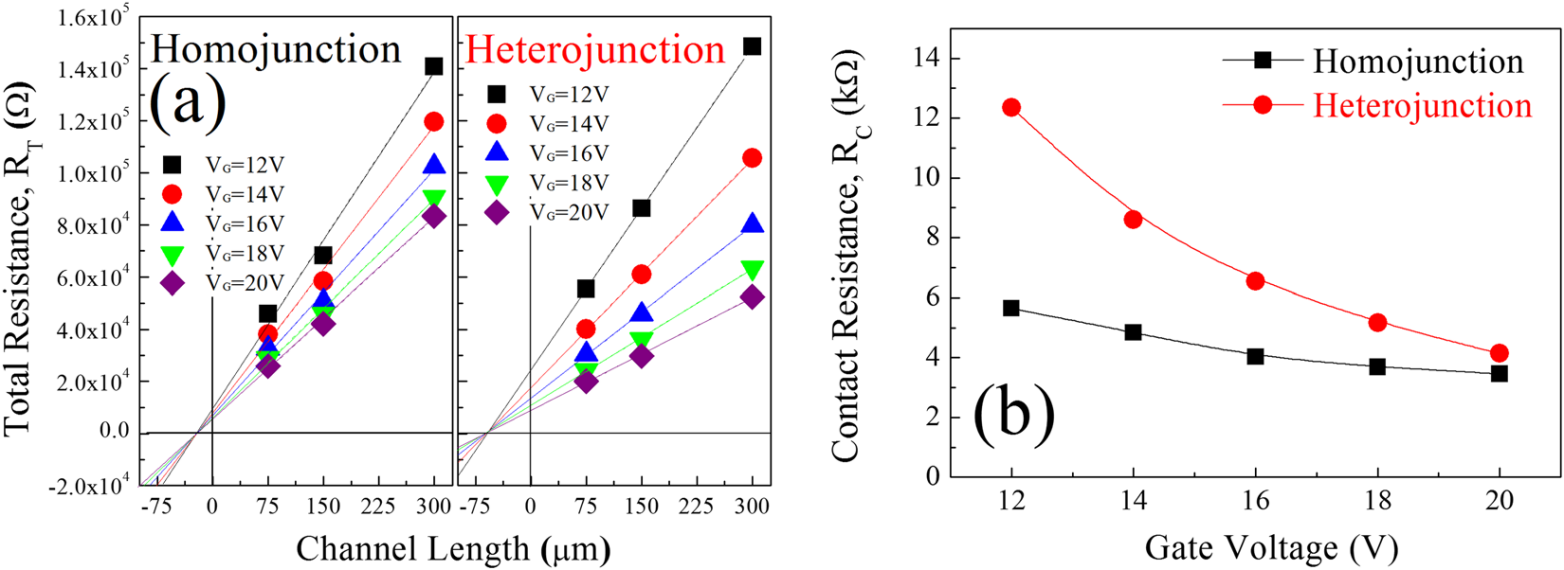
Electrical characterization of the WIZO-TFTs with different S/D electrodes. (**a**) Total resistance plotted against the channel length, allowing for extraction of contact resistance by the transmission line method (TLM). (**b**) Contact resistance plotted as a function of the gate voltage.

This enhancement of device performance may be attributed to lower contact resistance between the WIZO active-channel layer and conducting WIZO electrodes. Normally, contact resistance can be extracted by the TLM according to the following equation^25^:

1 $${R}_{T}=2RC+{r}_{ch}\cdot {L}_{eff},$$

of which *R*_*T*_ is total resistance, *R*_*C*_ is contact resistance, *r*_*ch*_ is semiconductor-channel resistance, and *L*_*eff*_ is effective-channel length. As shown in the equation, total resistance that is composed of contact and channel resistances was extracted from current-voltage curves at the WIZO-TFT device with various channel lengths (75 μm, 150 μm, and 300 μm). Here, the *r*_*ch*_ is a constant value because the homojunction-structured WIZO-TFT and the heterojunction-structured WIZO-TFT were fabricated on the same WIZO active layer; therefore, an *R*_*T*_ change according to a different S/D electrode means a change of the *R*_*C*_. Figure 2(a) shows the *R*_*T*_ versus the channel-length plot as a function of the gate voltage. To extract *R*_*C*_ values, we conducted linear fitting and intercept values of the *y*-axis when values of *x*-axis are zero as a function of the gate voltage was obtained as *R*_*C*_. Exhibited *R*_*C*_ of the WIZO-WIZO contact is lower than that of the WIZO-ITO contact as a function of the gate voltage, as shown in Fig. 2(b), thereby leading to transfer-characteristic differences. A low contact resistance of the homojunction-structured WIZO-TFT may be attributed to the ideal contact structure with identical oxide materials and absence of an interfacial layer.

**Figure 3:**
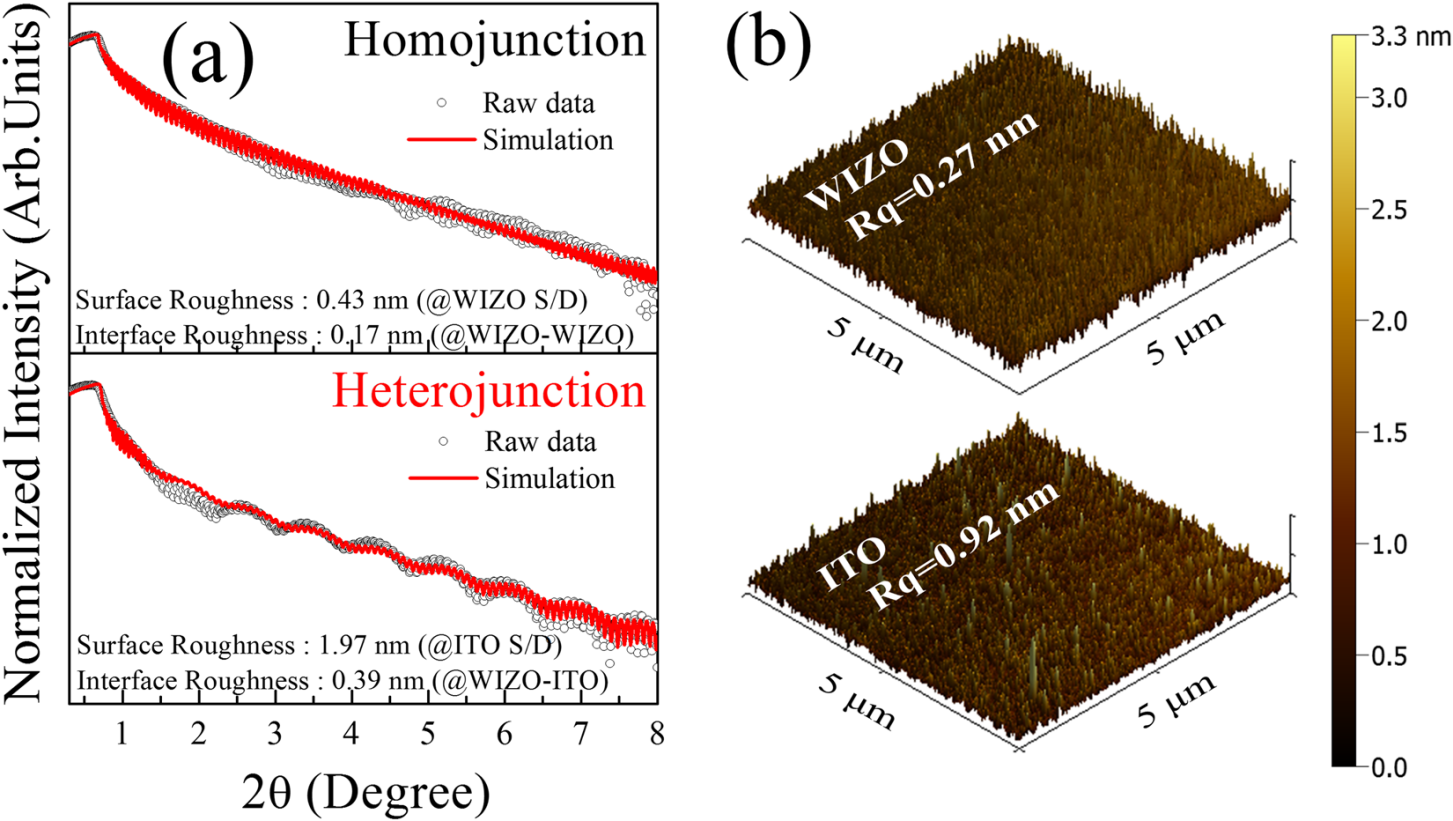
Physical properties of the WIZO film with the homojunction and the heterojunction. (**a**) Surface and interfacial roughness extracted from the XRR spectra. (**b**) Surface roughness of the WIZO and ITO films.

Figure 3(a) shows that measured XRR spectra and fitting results are in sound agreement, thereby validating the extracted XRR parameters. These oscillations are representations of interference of light reflected from various interfaces such as the air–S/D electrode, the S/D electrode–semiconductor active, the semiconductor active–insulator, and the insulator–substrate interface. Especially, to collect information on the interfacial layer according to different S/D electrode materials, the interfacial layer located between the S/D electrode and the WIZO active layer serves as the focus. First, film density, thickness, and roughness were analyzed for each single layer of the semiconducting WIZO layer, the conducting WIZO layer, and the ITO layer using the XRR analysis, as shown in S4 (Supplementary Information). Then, interfacial information of the multi-layer structure was obtained for the homojunction (WIZO-WIZO) and the heterojunction (WIZO-ITO) based on physical parameters extracted from each single layer. For the heterojunction, the surface- and interface-roughness values are 1.97 nm and 0.39 nm, respectively. Surface- and interface-roughness values of the homojunction of 0.43 nm and 0.17 nm, respectively, however, are more flat compared with the heterojunction. Results indicate that rough-interface correlation can interfere with electron transport that could be a plausible origin of degraded field-effect mobility of the heterojunction-structured WIZO-TFT. In addition, the surface roughness obtained by AFM analysis, revealed a similar tendency with XRR results, as shown in Fig. 3(b).

**Figure 4:**
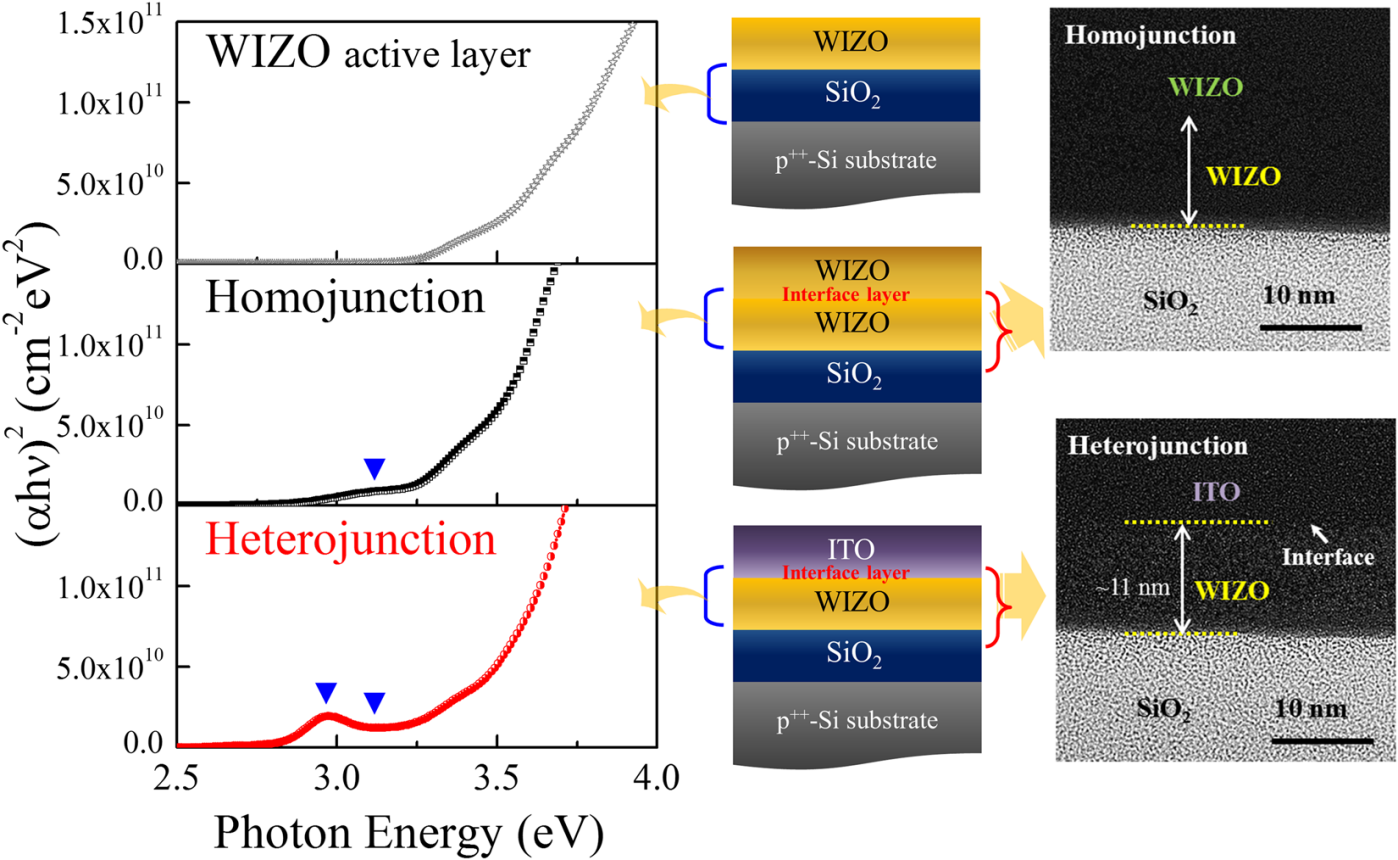
The three types of the absorption-coefficient spectra of the WIZO active layers such as the single WIZO layer and the WIZO layer affected by the homojunction and the heterojunction (left). Optical model for the extraction of the absorption-coefficient spectra (middle). Cross section images of the WIZO TFT according to the device structure (right).

**Figure 5:**
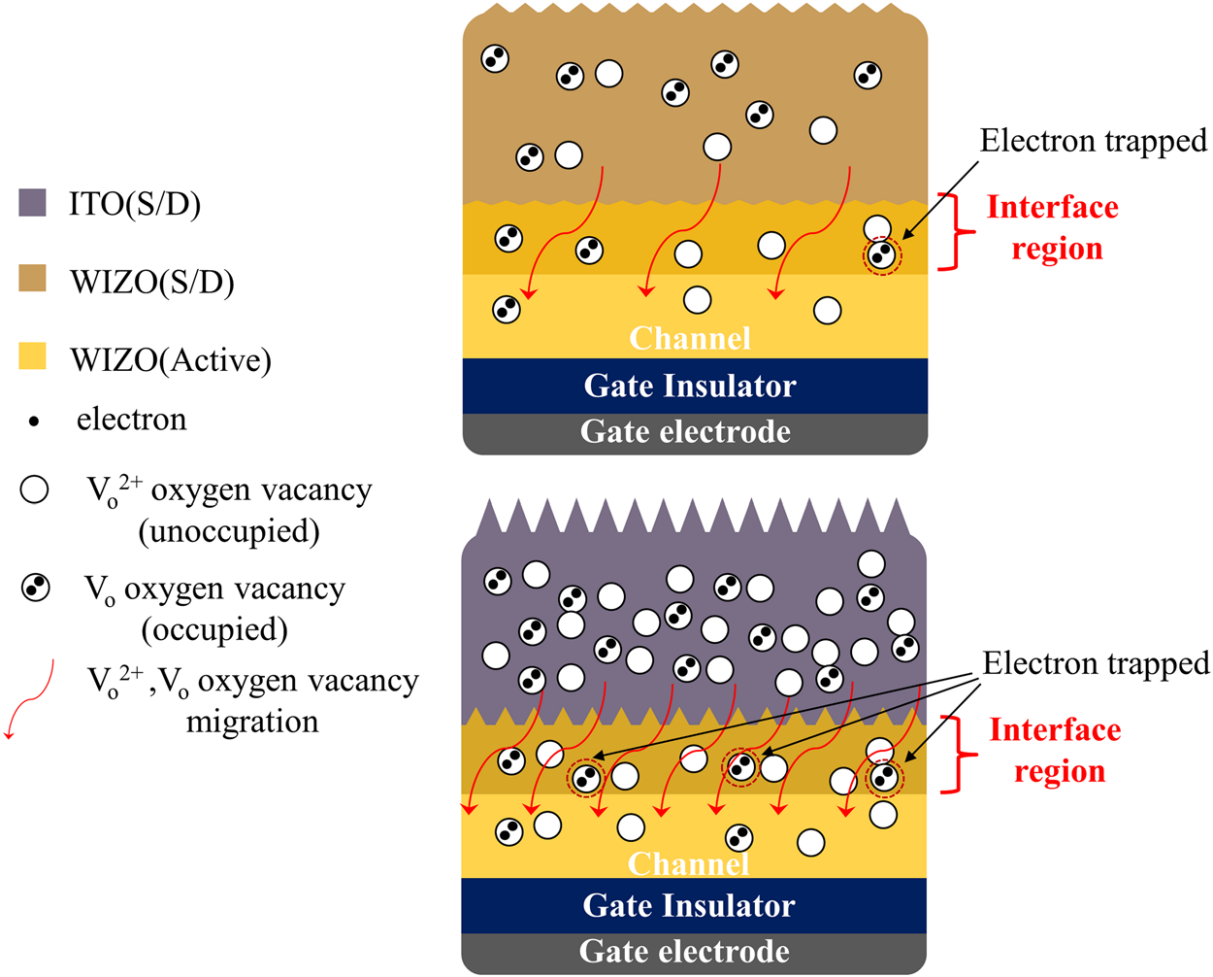
Schematic illustration of the electron-transport mechanism according to the S/D electrode materials.

The oxide semiconductor-active layer in which a channel is formed most significantly affects device performances of the oxide-TFT device. Also, since the stacked structure of the TFT devices consists of a semiconductor, an insulator, and an electrode, the active layer is influenced by the adjacent layer; therefore, to directly understand device performance of a TFT device, it is critical to analyze change of the electronic structure of the active layer that is caused by the adjacent layer with respect to the actual stacked structure. Figure 4 shows the three types of absorption-coefficient spectra of the WIZO active layers constituted of the single WIZO layer and the WIZO layers affected by the homojunction and the heterojunction. This spectrum of the single WIZO layer was extracted from the WIZO layer after establishing a four-phase optical model such as an actual multilayer situation, comprising of a Si substrate, a SiO_2_ layer, a WIZO layer, and an ambient layer. In particular, a detailed analysis of WIZO layer of the band edge states and unoccupied states in the conduction band was conducted by fitting using a Gaussian model (band edge states) and a Tauc-Lorentz model (conduction band states). In order to investigate change of the absorption coefficient of the WIZO layer according to the S/D electrode materials, a five-phase optical model was used by the insertion of an electrode layer between the semiconductor layer and the ambient layer. Regarding homojunction, the band-edge state below the conduction band of the WIZO active layer was slightly increased. The band-edge state of the WIZO active layer, however, was dramatically increased in the heterojunction. These changes of the band-edge state of the WIZO active layer according to S/D electrode materials may be attributed to hysteresis-derived degradation of device instability that may be strongly related to charge-trap densities, such as those of oxygen-related traps in the active layer and/or the electrode active-layer interface^26^. TEM images revealed that the interface region between the WIZO active layer and ITO electrode was obviously observed in the case of heterojunction structure, but no abruptly changed interface region was observed in homojunction structure. If the previous XRR, TEM and SE data are considered together, when the heterojunction-structured WIZO-TFT device is operated, electrons transferred from the S/D electrode to the active layer may be scattered due to the extent of interfacial roughness; the electrons may be trapped in electron-trap sites below the conduction band. In contrast, when the homojunction-structured WIZO-TFT device is operated, transport of electrons may easily occur due to the flat interfacial roughness and small amount of electron-trap sites, as shown in Fig. 5. Considering previous electrical data of device stability in Fig. 1, this result is strongly correlated with a rough interface, and a large number of electron-trap sites could be the plausible origin of degraded field-effect mobility and *S.S*. values that are caused by heterojunction.

**Figure 6:**
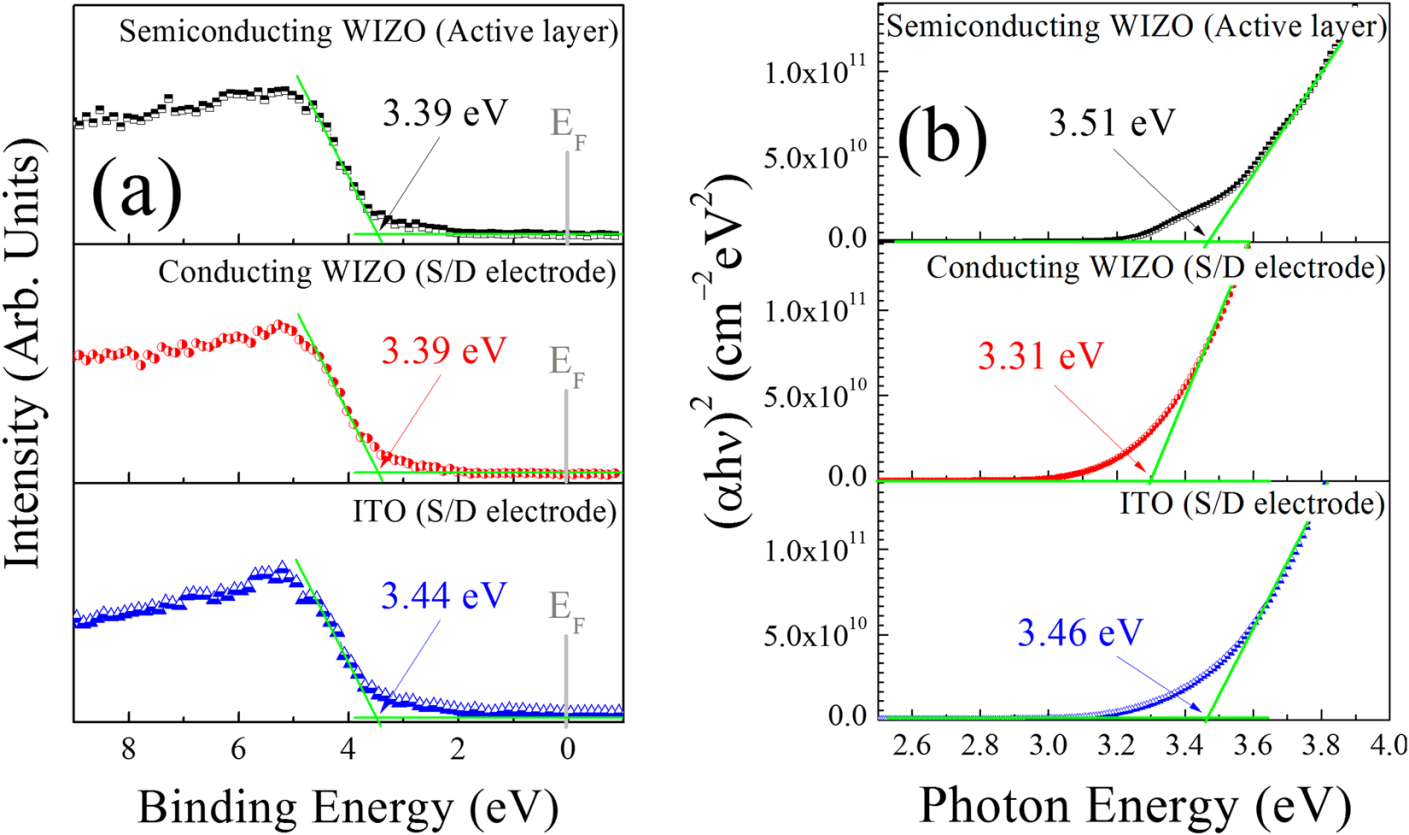
(**a**) Valence-band spectra and (**b**) optical bandgap of each of the single layers for the semiconducting WIZO, the conducting WIZO, and the ITO that were measured using the XPS and the SE according to the extrapolation method.

**Figure 7:**
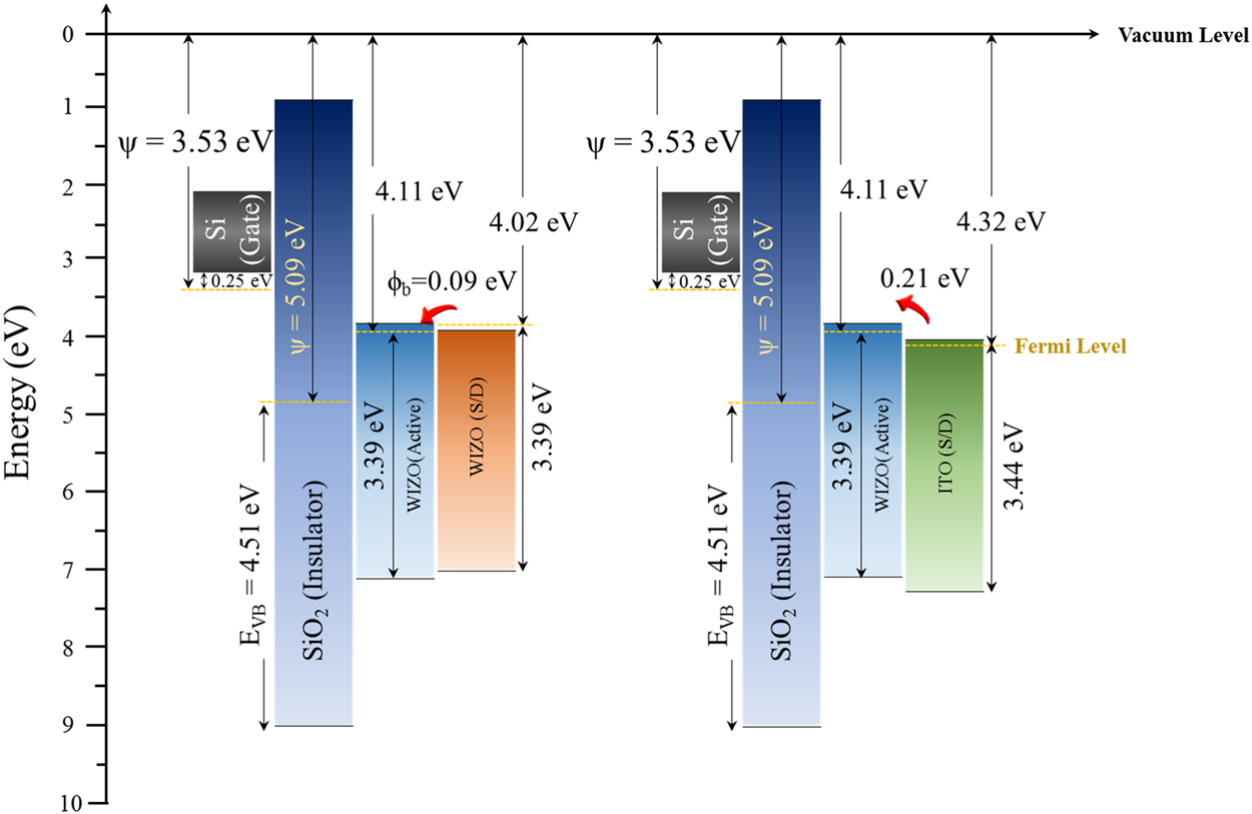
Schematic energy-level alignment reflecting the relative energy position of the Fermi level (E_F_) in terms of the conduction band minimum (CBM) and the valence-band maximum (VBM) with the homojunction and the heterojunction.

An understanding of the interfacial energy-level alignments between the active layer and the S/D electrode is indispensable for the design and realization of high-performance TFT devices. Therefore, for further discussion subsequently provided in this study, energy-level alignment between the Fermi level and the conduction-band minimum were considered. Figure 6(a) and (b) show the valence-band spectra and the bandgap of each single layer of the semiconducting WIZO layer, the conducting WIZO layer, and the ITO layer that were measured using XPS and SE according to the extrapolation method, respectively. Especially, the optical bandgap values were estimated from the Tauc-plot for direct-bandgap WIZO^27^. In addition, to conduct full energy-band alignment of adjacent-layer-constituting TFT devices, the valence-band spectra and the bandgap of the insulating SiO_2_ layer and the p^++^-Si substrate were analyzed again using the XPS and the SE, as shown in S5 (Supplementary Information). Through a combination of the spectral changes in Figs 6 and S5, energy-level alignments were evaluated and are shown in Fig. 7. The work function (${\rm{\Psi }}$) of each layer was obtained using KPFM. The electron barrier (${{\rm{\varphi }}}_{b}$) between the WIZO active layer and the conducting WIZO electrode of the homojunction is 0.09 eV, that is slightly smaller than the 0.21 eV electron barrier between the WIZO active layer and the conducting ITO electrode of the heterojunction. These reduced electron barriers may be attributed to enhancement of electrical performance of the TFT device that is due to easy transference of electrons injected from the S/D electrode to the active-channel layer.

## Conclusion

In conclusion, homojunction- and heterojunction-structured WIZO-TFTs were fabricated using two types of S/D electrode material, the conducting WIZO and ITO, on the same WIZO active-channel layer. The homojunction-structured WIZO-TFT exhibited an excellent device performance including saturation mobility of 26.10 cm^2^/Vs, an *S.S*. value of 0.38 V/decade, and a hysteresis-based *ΔV*_*th*_ that is within 0.07 V. In contrast, in the case of the heterojunction-structured WIZO-TFT, saturation mobility, *S.S*. value, and hysteresis-based *ΔV*_*th*_ were degraded to 21.02 cm^2^/Vs, 0.44 V/decade, and 0.32 V, respectively. Enhancement of device performance of the WIZO-TFT with the homojunction structure originated from the flat interfacial roughness and a small number of electron-trap sites. The electron barrier of the homojunction-structured WIZO-TFT is 0.09 eV, which is lower than the value of 0.21 eV that was obtained for the heterojunction-structured WIZO-TFT. This reduced electron barrier may be attributed to enhancements of device performance and stability, that are related to the carrier transport. Results suggest that control of the interfacial status is significant in the fabrication of high-performance TFT devices.

## Electronic supplementary material

supplementary information
