# Supplementary material for: Enhancement of the Device Performance and the Stability with a Homojunction-structured Tungsten Indium Zinc Oxide Thin Film Transistor
Source: Sci Rep. 2017 Sep 14;7:11634. doi: 10.1038/s41598-017-12114-y (PMC5599534; doi:10.1038/s41598-017-12114-y)
Supplement: Supplementary file 1 — supplementary information [file 41598_2017_12114_MOESM1_ESM.doc]

Enhancement of the Device Performance and the Stability with a Homojunction-structured Tungsten Indium Zinc Oxide Thin Film Transistor

Hyun-Woo Park ^1,2^, Aeran Song^1^, Dukhyun Choi ^2^, Hyung-Jun Kim^3^, Jang-Yeon Kwon^3^, Kwun-Bum Chung ^1^,*[[1]](#footnote-2)*

^1^Division of Physics and Semiconductor Science, Dongguk University, Seoul, 100-715, Korea

^2^Department of Mechanical Engineering, School of Engineering, Kyung Hee University, Yongin, 446-701, Korea

^3^Yonsei Institute of Convergence Technology, Yonsei University, Incheon, 406-840, Korea

***Supplementary figure S1****. Electrical property of WIZO TFTs and optical bandgap of WIZO films as a function of oxygen partial pressure during the sputtering deposition process*

*
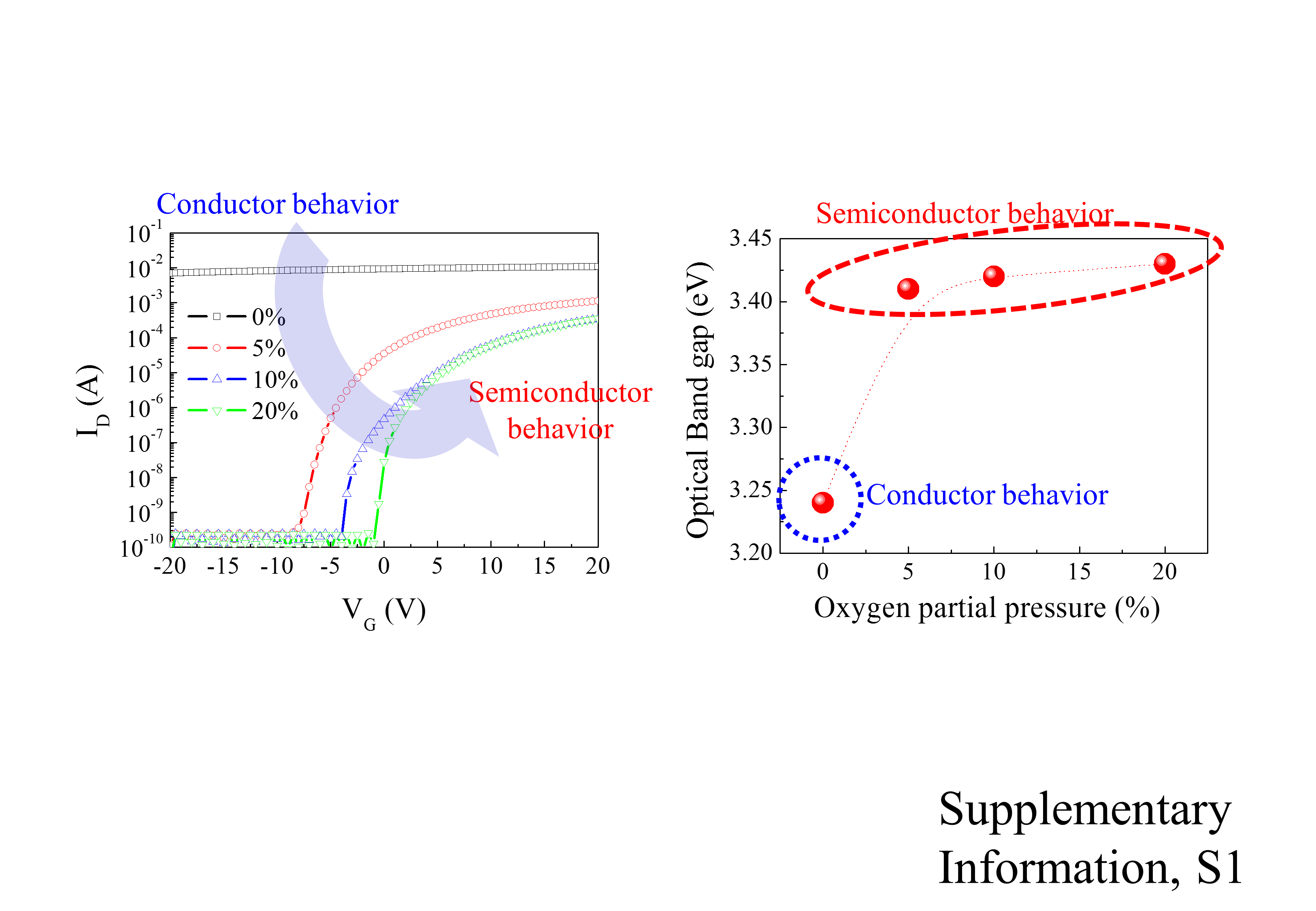
*


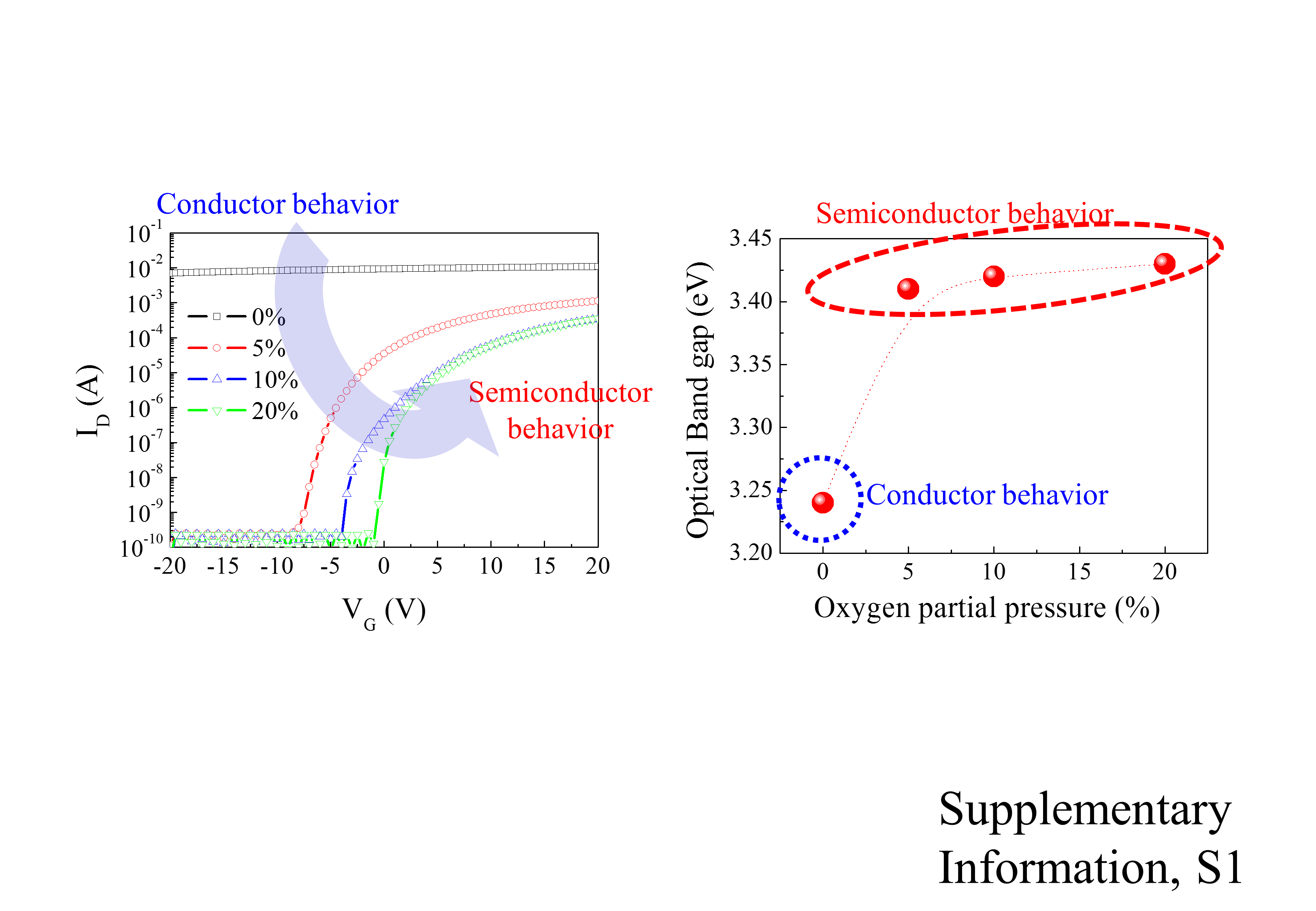


***Supplementary figure S2****. Physical structure and chemical composition of WIZO films as a function of oxygen partial pressure (P_O2_) during the deposition process*

***
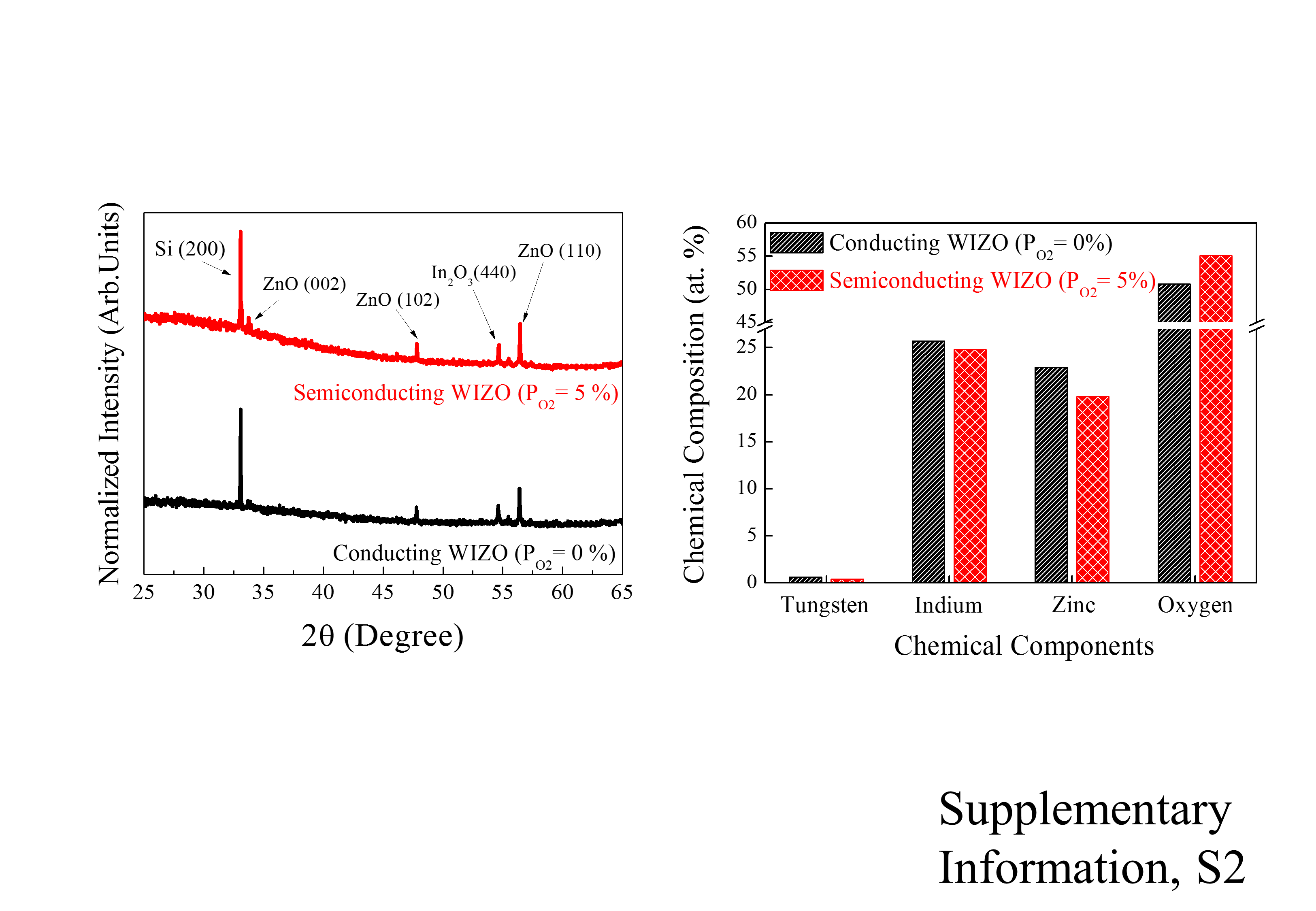
***

***
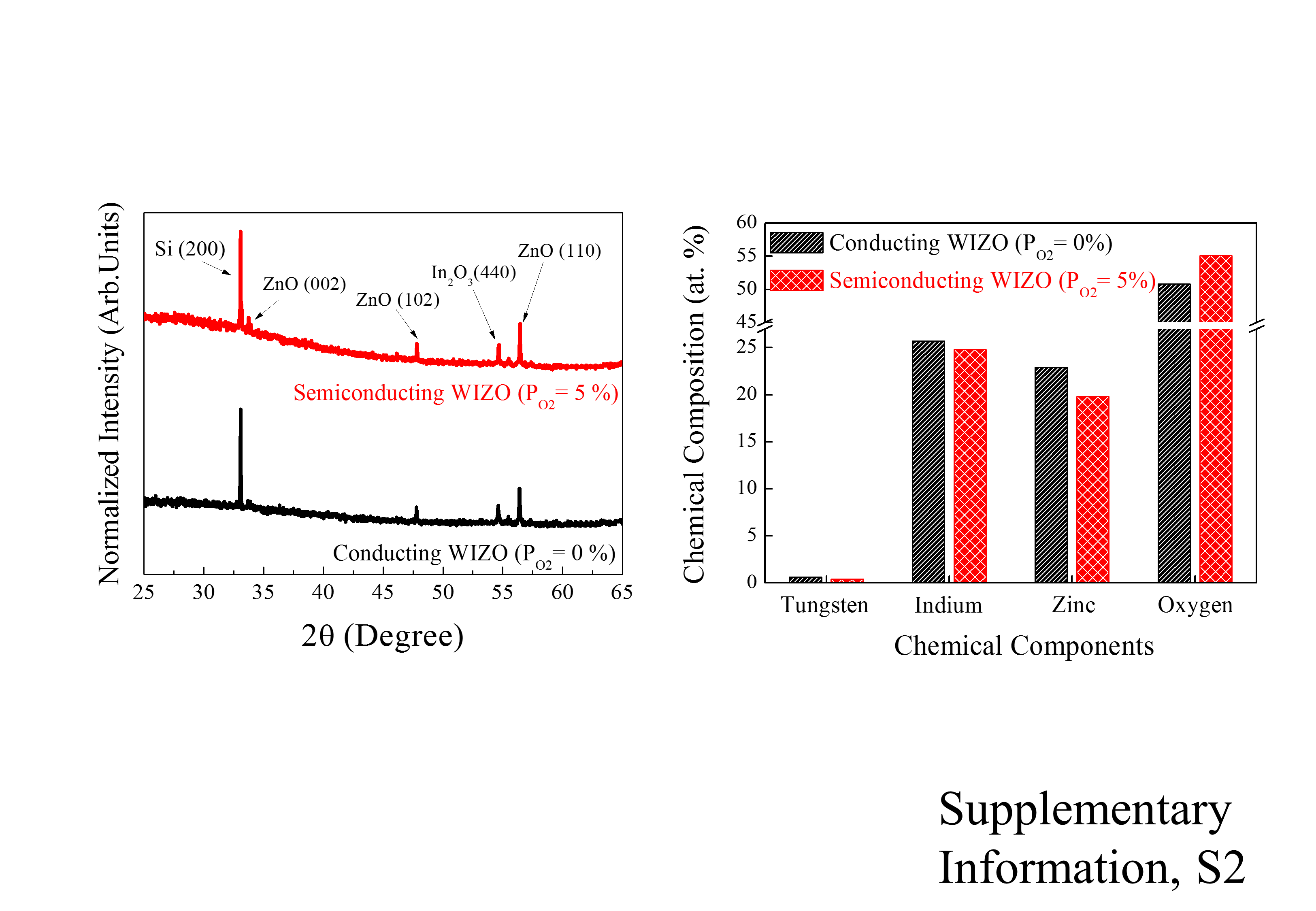
***

***Supplementary figure S3****. Electrical property of homojunction structured WIZO TFTs and conducting WIZO films as a function of W doping concentration for W doping amount optimization*

*
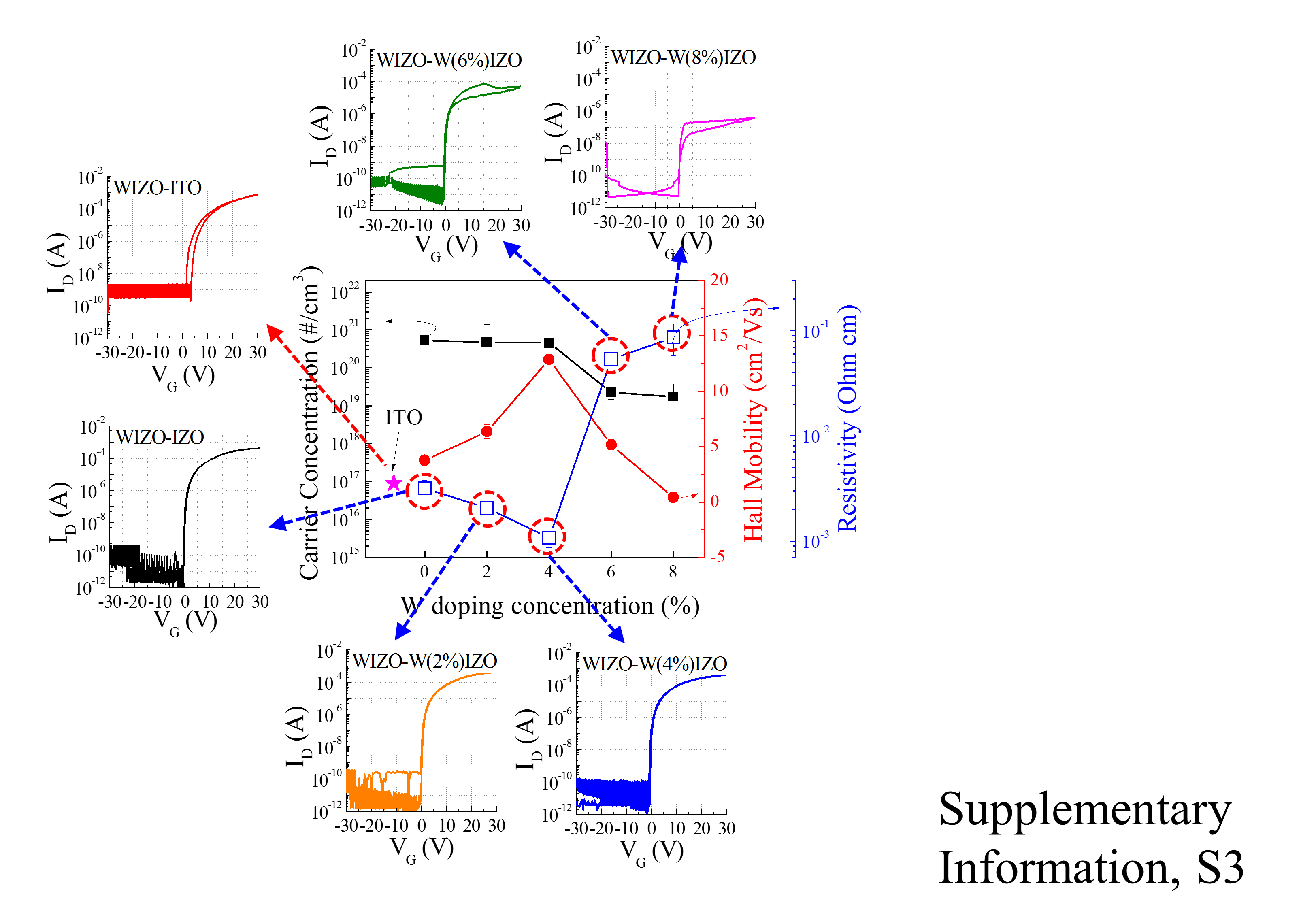
*

***Supplementary figure S4****. Film density, thickness, and surface roughness were analyzed by XRR analysis for each single layer of semiconducting WIZO layer, conducting WIZO layer, and ITO layer*

***Supplementary figure S5****. Valence band spectra and bandgap of insulating SiO_2_ layer and p^++^-Si substrate measured by XPS and SE using the extrapolation method, respectively*

*
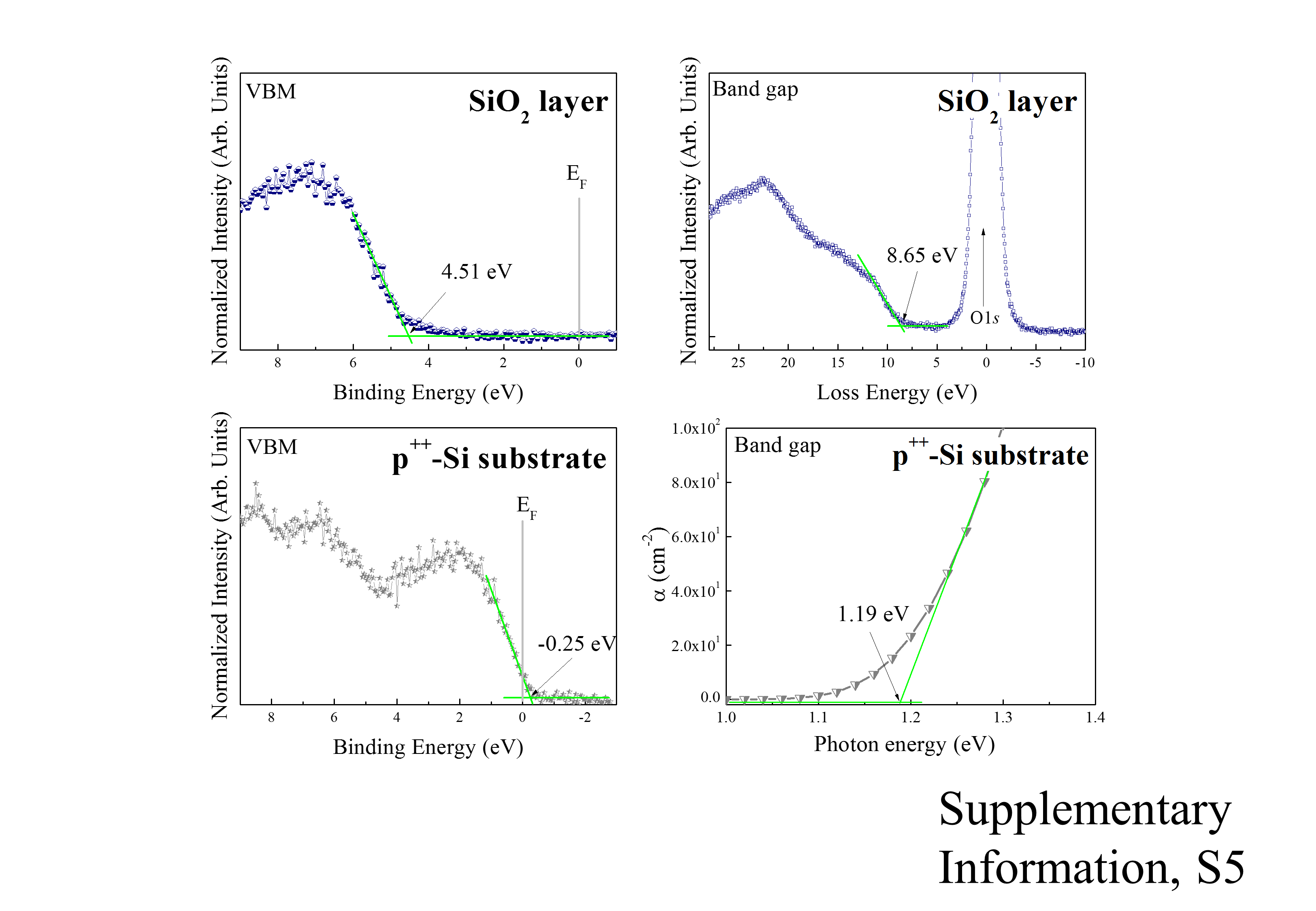
*

1. e-mail of corresponding Author: kbchung@dongguk.edu (Kwun-Bum Chung) [↑](#footnote-ref-2)
